# Cardiovascular risk scores in asymptomatic carotid stenosis: A validation study with ultrasonographic parameters

**DOI:** 10.1371/journal.pone.0265732

**Published:** 2022-04-01

**Authors:** Min Kyoung Kang, Ki-Woong Nam, Jung Hwan Shin, Hyung-Min Kwon, Yong-Seok Lee

**Affiliations:** 1 Department of Neurology, Uijeongbu Eulji Medical Center, Uijeongbu, Republic of Korea; 2 Department of Neurology, Seoul Metropolitan Government Seoul National University Boramae Medical Center, Seoul, Republic of Korea; 3 Department of Neurology, Seoul National University College of Medicine, Seoul, Republic of Korea; IPATIMUP/i3S, PORTUGAL

## Abstract

We evaluated the feasibility of the Framingham stroke risk score (FSRS) and atherosclerotic cardiovascular disease (ASCVD) risk scores for asymptomatic carotid stenosis (ACS). In addition, we developed novel risk prediction models for ischemic stroke and composite outcomes by combining ultrasonographic parameters and conventional cardiovascular risk scores. We retrospectively enrolled 612 patients with ACS greater than 50% over 7 years and evaluated them using transcranial Doppler and carotid duplex ultrasonography. In total, 150 patients were included in the analysis. During the mean 5-year follow-up, 6 ischemic strokes and 25 composite events were detected. Among all ultrasonographic parameters, only a higher peak-systolic velocity/end-diastolic velocity ratio was detected and significantly associated with an increased risk of relevant ischemic stroke (hazard ratio: 1.502, 95% confidence interval: 1.036–1.968). The C-statistics of the FSRS and ASCVD risk scores were 0.646 and 0.649, respectively, for relevant ischemic stroke, and 0.612 and 0.649, respectively, for composite outcomes. C-statistics of the FSRS and ASCVD risk scores combined with ultrasonographic parameters increased to 0.937 and 0.941, respectively, for ischemic stroke, and 0.856 and 0.886, respectively, for composite outcomes. The study suggests that inclusion of ultrasonographic parameters in conventional cardiovascular scores helps identify the risk of further vascular events in ACS patients.

## Introduction

The Framingham risk score (FRS) and atherosclerotic cardiovascular disease (ASCVD) risk scores are widely used in the stratification of high-risk patients for cardiovascular disease (CVD) or stroke in patients with a cardiovascular risk factor [1–3]. The FRS and ASCVD risk scores, which were initially pooled from cohort participants between 1965 and 1995 for risk prediction and effective management of CVD, have been validated in diverse cohorts. Our previous studies have also confirmed that intracranial atherosclerosis and extracranial atherosclerosis are closely related to high ASCVD scores [4]. However, they have not been validated in symptomatic cerebrovascular disease in asymptomatic carotid stenosis (ACS), which is one of the major causes of stroke [5].

ACS is found in 4.2% of the general population, and its prevalence increases with age [6]. Since the annual stroke rate in ACS ranges from 1% to 3%, the prediction of ischemic stroke (IS) in patients with ACS is important in clinical practice [7]. However, previous studies could not provide sufficient information to evaluate the risk of stroke in ACS [8]. Therefore, it is crucial to establish a prediction model that can identify high-risk patients with ACS.

Ultrasonography is widely preferred in clinical practice for patients with ACS due to its noninvasive nature and ability to provide rich and objective information on cerebral hemodynamics that account for the mechanism of stroke. Common carotid artery intima-media thickness (CIMT) and plaques are useful ultrasonographic markers of asymptomatic and subclinical atherosclerosis [9–12]. However, the sole introduction of these markers could not provide solid evidence for improving the performance of the FRS or ASCVD scores [13, 14]. In addition to these two factors, we have introduced hemodynamic ultrasound parameters to enhance the accuracy of conventional cardiovascular risk scores.

Considering the need for prediction models to identify high-risk patients with ACS, we aimed to validate the conventional cardiovascular risk scores for ACS and develop novel risk prediction models for ischemic stroke and composite outcomes of IS, transient ischemia attack (TIA), CVD, peripheral artery disease (PAD), and mortality from CVD in ACS by combining ultrasonographic parameters and conventional cardiovascular risk scores.

## Methods

### Study subjects

From January 2010 to December 2017, we retrospectively enrolled consecutive 612 Korean patients with carotid stenosis >50% using the carotid duplex ultrasonography (CDU) criteria. The medical records of the patients were reviewed to assess conventional risk scores (Framingham stroke risk score [FSRS] and ASCVD risk score) and to confirm the occurrence of IS, TIA, CVD, PAD, and mortality from CVD for up to 10 years. We excluded patients with a history of existing symptomatic ASCVD, including IS, TIA, CVD, PAD, treatment with invasive procedures, non-atherosclerotic carotid stenosis, and missing data for baseline covariates or magnetic resonance imaging or angiography, as shown in S1 Fig. In total, 150 patients were included in the analysis. For these patients, the FSRS and ASCVD risk scores were calculated using baseline data [1, 15]. This study was approved by the Institutional Review Board of Seoul Metropolitan Government-Seoul National University Boramae Medical Center with waiver of documentation of consent and performed in accordance with relevant guidelines and regulations (number: 30-2019-89).

### Measurements detailed

At the first outpatient visit, the systolic blood pressure (SBP) and diastolic blood pressure (DBP) of the patients were determined with a mercury sphygmomanometer with at least 10 minutes of relaxation, using standard procedures [16]. Serum levels of total cholesterol and high-density lipoprotein (HDL) were measured using enzymatic assay after at least 8-hours of fasting [17]. Subjects were considered to be diabetic if they reported a medical history of diabetes mellitus (DM) or use of antidiabetic drugs or if they met the criteria for the diagnosis of DM [18]. History of hypertension and prior CVD was defined using subject self-report, medical records, or current use of medications. Atrial fibrillation and left ventricular hypertrophy (LVH) were defined using subject self-reports, medical records, and electrocardiograms. A smoking history was defined as cigarette smoking. The FSRS was calculated using the following variables: age, sex, SBP, total cholesterol, HDL, treatment of hypertension, history of DM, CVD, cigarette smoking, atrial fibrillation, and LVH [15]. The ASCVD risk score was calculated using the following variables: age, race, sex, SBP, DBP, total cholesterol, HDL, hypertension, history of DM, and cigarette smoking [1].

### Ultrasonographic examinations

The initial results of ultrasonographic examination of the patient was used in the prediction model. Two experienced sonographers at our center performed the ultrasonographic examinations throughout the study period. Transcranial Doppler ultrasonography (TCD) was recorded using standardized scanning protocols as previously reported [19]. In brief, the physiological data of the mean flow velocities (MFV) and the pulsatility index (PI) were obtained from insonation of the transtemporal approach for the middle cerebral artery (MCA), anterior cerebral artery and posterior cerebral artery; the transorbital approach for the ophthalmic artery (OA) and internal carotid artery (ICA); and the transforaminal approach for both vertebral and basilar arteries. Collateral circulation was determined by inspection of the OA, anterior communicating artery (Acom), or posterior communicating artery (Pcom) [20]. CDU was recorded at the common carotid artery (CCA) to the ICA, including the proximal and distal parts of the maximal stenosis site. The CIMT and plaque measurements were performed by Mannheim consensus [21]. In brief, a double-line pattern observed at the far wall of the CCA, at least 5 mm below its end on a longitudinal image, was defined as CIMT. Plaques were defined as focal structures encroaching into the arterial lumen of at least 0.5 mm or 50% of the surrounding intima-media thickness value or a thickness > 1.5 mm as measured from the intima-lumen interface to the media-adventitia interface. The number, location, form, surface, echogenicity, and texture of each plaque were described as previously reported [22]. We evaluated the additional hemodynamic status in CDU as the primary parameter of peak-systolic velocity (PSV) of the ICA and CCA and end-diastolic velocity (EDV) of the CCA by recording the PSV and EDV in the distal CCA within 2 cm of the bifurcation and in the ICA at the location where the highest PSV was observed. We calculated the secondary parameters of the PSV (PSV_ICA_/PSV_CCA_) ratio and PSV/EDV (PSV_ICA_/EDV_CCA_) ratio [23, 24]. The hemodynamic criterion adopted for the determination of stenosis degree included the ICA PSV, PSV ratio, and PSV/EDV ratio [23, 25].

### Statistical analysis

Novel risk prediction models that combined conventional cardiovascular risk scores and ultrasonographic parameters were developed with all possible combinations of ultrasonographic parameters, and the three most highly achieved models of more than 500 combination models under Cox regression methods were presented. The primary outcome of relevant IS was defined as IS and TIA in the ipsilateral hemisphere. Composite outcomes were obtained from IS, TIA, CVD, PAD, and mortality from CVD and stroke. Continuous variables were expressed as either mean values (standard deviation) or median values (with interquartile range), as appropriate. Categorical variables were expressed as proportions. Cox regression analysis was performed to validate the performance of the conventional cardiovascular risk scores in predicting outcomes in ACS. Assessment of each novel risk prediction model of adjunction of ultrasonographic parameters to conventional risk scores was performed by receiver operating characteristics (ROC) curve comparison.

The level of statistical significance was set at P<0.05. All statistical analyses were performed using SPSS software (version 25.0, SPSS Inc., Chicago, IL, USA), MATLAB (version 2019a, Mathworks Inc., Natwick, MA, USA) and SAS 9.4 software (SAS Studio 3.7, SAS Institute, Cary, North Carolina, USA) with two-sided significance set at 0.05.

## Results

### Baseline characteristics

The baseline demographic and clinical characteristics of the subjects are presented in Table 1. The 25^th^ and 75^th^ percentiles of risk scores were 11.5% and 30.8% for FSRS and 18.6% and 42.6% for the ASCVD risk scores, respectively. When evaluated using ultrasonographic parameters, collateral flow was observed in the OA (n = 10), Acom (n = 13), Pcom (n = 1), or more than one artery (n = 9). CIMT increased more than 1.1 mm in 52 patients (34.6%) in the overall population. Three patients with normal PSVs met secondary parameters of carotid artery stenosis. Over a mean follow-up period of 5 years, 6 patients had relevant IS, 12 had IS or TIA, 10 had definite or probable CVD, and 4 had PAD confirmed through symptoms, laboratory examination, and relevant imaging. Three deaths were attributed to CVD and one to stroke.

**Table 1:** Baseline characteristics of included patients.

	Entire subjects (N = 150)
**Age, mean (SD), year**	65.56 ± 6.14
**Male, n (%)**	113 (75.3)
**Systolic blood pressure, mean (SD), mmHg**	143.19 ± 21.43
**Diastolic blood pressure, mean (SD), mmHg**	77.22 ± 13.15
**Total cholesterol, mean (SD), mg/dl**	164.39 ± 35.78
**HDL cholesterol, mean (SD), mg/dl**	45.49 ± 11.58
**Hypertension, n (%)**	123 (82.0)
**Diabetes Mellitus, n (%)**	56 (37.3)
**Cigarette smoking, n (%)**	42 (28.0)
**Atrial fibrillation, n (%)**	8 (5.3)
**Left ventricular hypertrophy, n (%)**	31 (20.7)
**Conventional Risk Scores**	
** 10-year Framingham stroke risk, median [IQR], %**	16.3 [11.5–30.8]
** 10-year Atherosclerotic Cardiovascular Disease risk, median [IQR], %**	33.4 [18.6–42.6]
**Sonographic parameters**	
** *Transcranial Doppler***	
** MFV of MCA, mean (SD), cm/s**	58.3 (27.0)
** PI of MCA, mean (SD),**	0.87 (0.29)
** Presence of collateral flow, n (%)**	33 (22.0)
** *Carotid Duplex Ultrasonography***	
** CIMT, mean (SD), mm**	0.98 (0.34)
** Ulcerative or Hypoechoic plaque, n (%)**	20 (13.3)
** PSV of ICA, mean (SD), cm/s**	194.0 (83.3)
** PSV of CCA, mean (SD), cm/s**	61.2 (20.5)
** EDV of CCA, mean (SD), cm/s**	14.3 (3.8)
** PSV ratio, mean (SD)**	3.2 (2.3)
** PSV/EDV ratio, mean (SD)**	15.2 (11.4)
**Outcome Events**	
** Follow-up period, median [IQR], year**	5 [3–7]
** Relevant ischemic stroke, n (%)**	6 (4.0)
** Any ischemic stroke/TIA, n (%)**	12 (8.0)
** Cardiovascular events, n (%)**	10 (6.7)
** Peripheral artery events, n (%)**	4 (2.6)
** Cardiovascular and stroke-related death, n (%)**	4 (2.6)
** Composite outcomes, n (%)**	25 (18.0)

Abbreviations: SD, standard deviation; HDL, high-density lipoprotein; IQR, interquartile range; MFV, mean flow velocity; MCA, middle cerebral artery; PI, pulsatility index; CIMT, carotid intima-media thickness; PSV, peak-systolic velocity; ICA, internal carotid artery; CCA, common carotid artery; EDV, end-diastolic velocity; PSV ratio, PSV_ICA_/PSV_CCA_; PSV/EDV ratio, PSV_ICA_/EDV_CCA_; TIA, transient ischemic attack.

### Factors related to cerebrovascular risk and composite outcome prediction

In the univariate Cox regression analysis for identifying the risk factors of relevant IS, only the PSV/EDV ratio was statistically significant (Table 2); the FSRS and ASCVD risk score were not statistically significant (p = 0.061 and 0.109, respectively). However, the FSRS and ASCVD risk scores were independently associated with composite outcomes (hazard ratio [HR] 1.019, 95% confidence interval [CI] 1.003–1.036, p = 0.021; HR 1.030, 95% CI 1.009–1.053, p = 0.006, respectively). In addition, the PI of the MCA was also associated with composite outcomes (HR 4.343, 95% CI 1.005–18.053, p = 0.043). In the multivariate Cox regression analysis of ASCVD risk score combined with PI for composite outcomes, only the ASCVD risk score was statistically significant (HR 1.031, 95% CI 1.006–1.056, p = 0.012). However, in the multivariate analysis of FSRS and PI, FSRS was marginally significant (HR 1.017, 95% CI 0.999–1.036, p = 0.052).

**Table 2:** Factors associated with the relevant ischemic stroke or composite outcomes.

	Relevant Ischemic Stroke		Composite outcomes	
	Unadjusted HR (95% CI*)	P-value	Unadjusted HR (95% CI*)	P-value
**FSRS**	1.061 (0.997–1.129)	0.061	1.019 (1.003–1.036)	0.021*
**ASCVD risk score**	1.079 (0.983–1.185)	0.109	1.030 (1.009–1.053)	0.006*
**MFV of MCA**	1.016 (0.970–1.063)	0.501	0.989 (0.975–1.004)	0.989
**PI of MCA**	1.156 (0.876–1.436)	0.558	4.343 (1.005–18.053)	0.043*
**Collateral flow in TCD**	2.657 (0.165–42.870)	0.491	1.273 (0.590–2.746)	0.539
**CIMT**	1.028 (0.802–1.254)	0.718	1.329 (0.402–4.394)	0.641
**Ulcerative or Hypoechoic plaque**	0.040 (0.000–21.931)	0.723	0.826 (0.290–2.354)	0.721
**PSV of ICA**	1.001 (0.986–1.016)	0.893	1.002 (0.998–1.007)	0.353
**PSV ratio**	1.042 (0.589–1.844)	0.888	1.091 (0.938–1.268)	0.258
**PSV/EDV ratio**	1.502 (1.036–1.968)	0.034*	1.022 (0.992–1.053)	0.146

Abbreviations: HR, hazard ratio; CI, confidence interval; FSRS, Framingham Stroke Risk Score; ASCVD, atherosclerotic cardiovascular disease; MFV, mean flow velocity; MCA, middle cerebral artery; PI, pulsatility index; TCD, transcranial Doppler ultrasonography; CIMT, carotid intima-media thickness; PSV, peak-systolic velocity; ICA, internal carotid artery; PSV ratio, PSV_ICA_/PSV_CCA_; PSV/EDV ratio, PSV_ICA_/EDV_CCA._

* indicates a significant difference (p<0.05).

### Validation of conventional cardiovascular risk scores and improvement of prediction power using combination with ultrasonographic parameters

**Fig 1:**
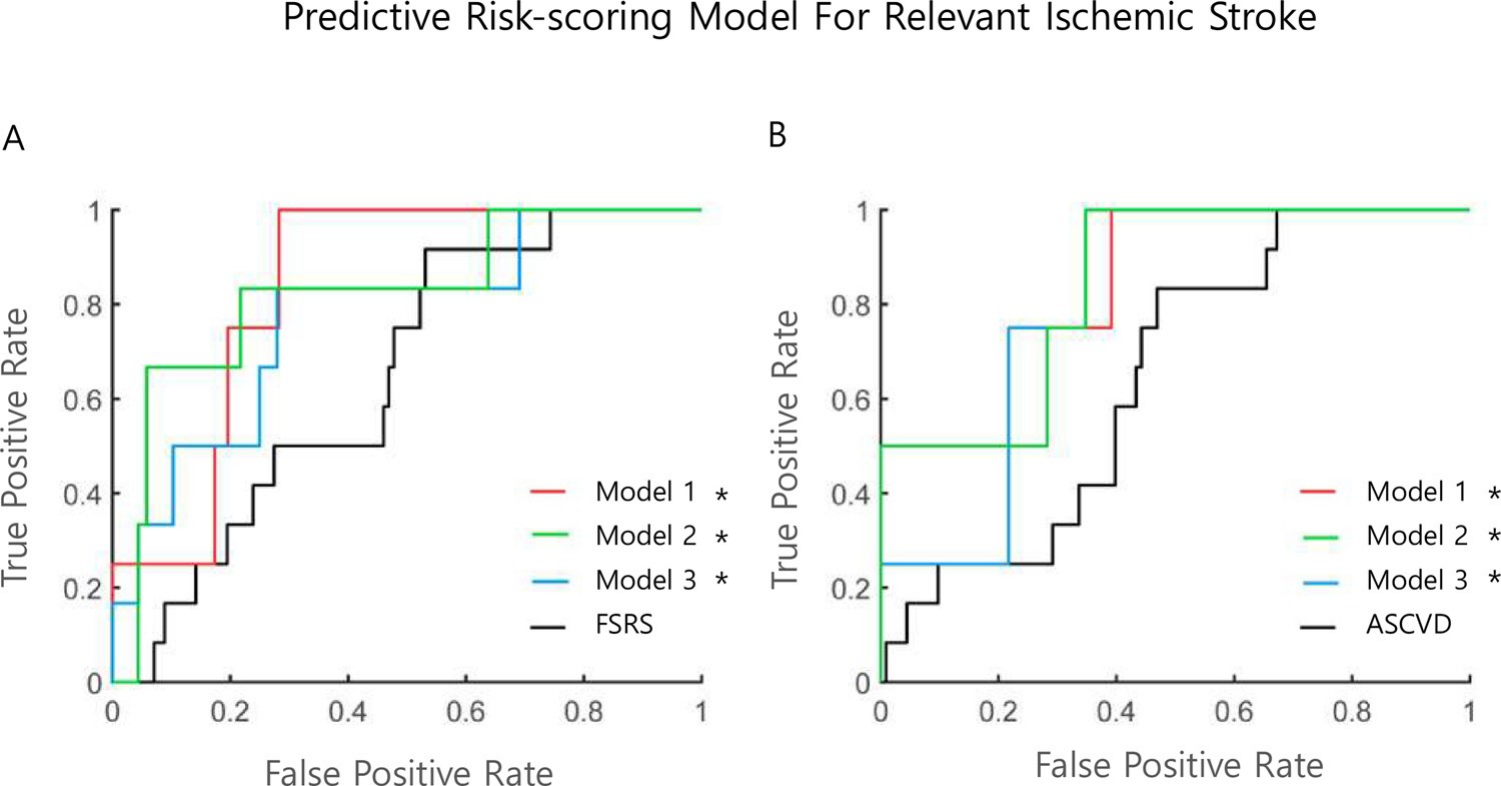
Comparison of ROC curves of novel risk prediction models for relevant ischemic stroke. A. Different models based on the FSRS for prediction of relevant ischemic stroke. ROC curves in the FSRS (black line), Model 1 (FSRS, PSV, CIMT, high-risk plaque, and PI, red line), Model 2 (FSRS, PSV ratio, CIMT, high-risk plaque, and PI, green line), and Model 3 (FSRS, PSV/EDV ratio, CIMT, high-risk plaque, and PI, blue line) are shown. B. Different models based on the ASCVD risk score for prediction of relevant ischemic stroke. ROC curves in ASCVD risk score (black line), Model 1 (ASCVD, PSV, CIMT, high-risk plaque, and PI, red line), Model 2 (ASCVD, PSV/EDV ratio, CIMT, high-risk plaque, and PI, green line), and Model 3 (ASCVD, PSV ratio, CIMT, high-risk plaque, and PI, blue line) are shown. Abbreviations: ROC, receiver operating characteristics; FSRS, Framingham stroke risk score; PSV, peak-systolic velocity; CIMT, common carotid artery intima-media thickness; PI, pulsatility index; PSV/EDV ratio, peak-systolic-and-end-diastolic-velocity-ratio; ASCVD, atherosclerotic cardiovascular disease. *indicates statistically significant (p<0.05) differences relative to classical cardiovascular risk scores (black lines).

**Fig 2:**
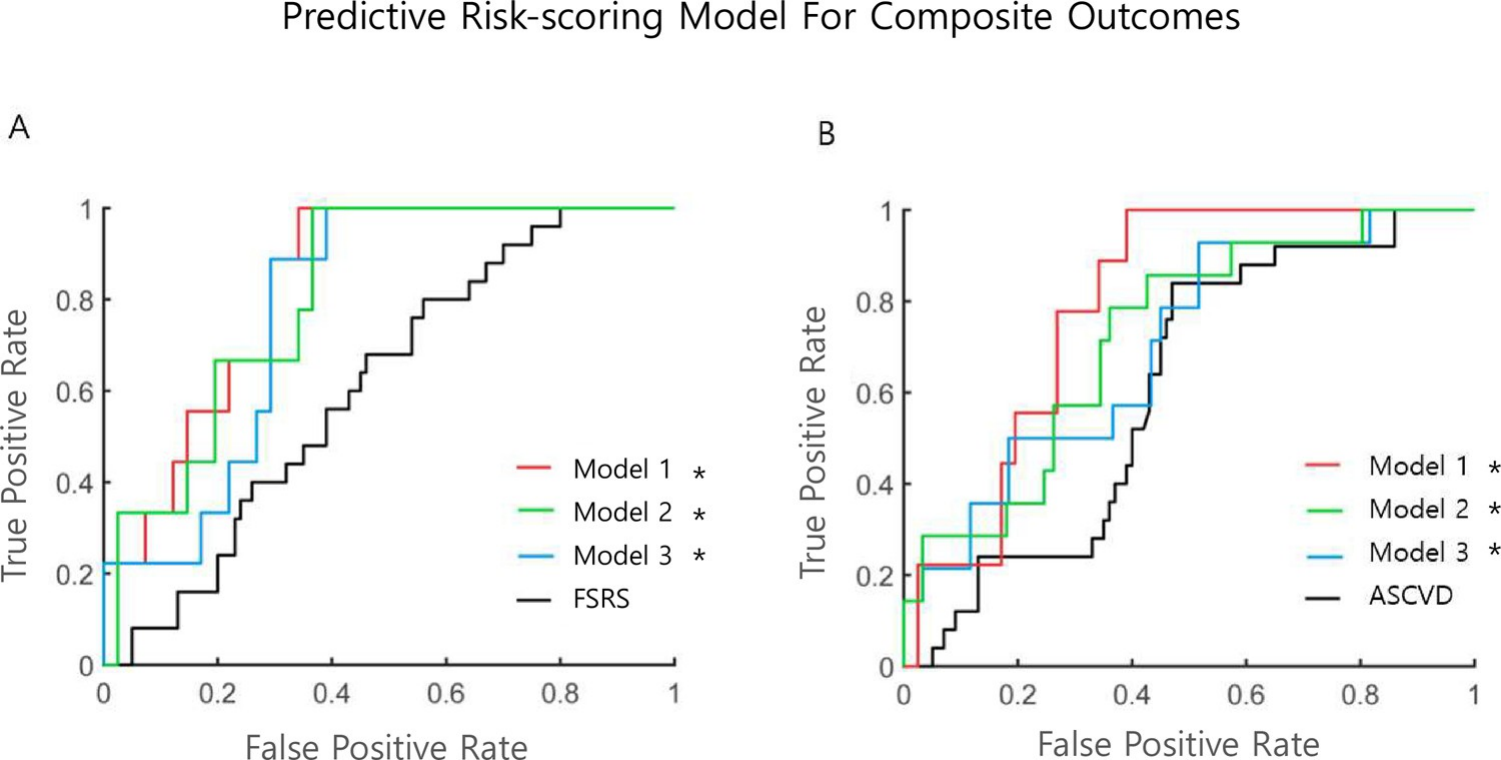
Comparison of ROC curves of novel risk prediction models for composite outcomes. A. Different models based on the FSRS for prediction of composite outcomes. ROC curves in the FSRS (black line), Model 1 (FSRS, PSV ratio, CIMT, high-risk plaque, collateral flow, MFV, and PI, red line), Model 2 (FSRS, PSV/EDV ratio, CIMT, high-risk plaque, collateral flow, MFV, and PI, green line), and Model 3 (FSRS, PSV, CIMT, high-risk plaque, collateral flow, and PI, blue line) are shown. B. Different models based on the ASCVD risk score for prediction of composite outcomes. ROC curves in the ASCVD risk score (black line), Model 1 (ASCVD, PSV, CIMT, high-risk plaque, and PI, red line), Model 2 (ASCVD, PSV/EDV ratio, CIMT, MFV, and PI, green line), and Model 3 (ASCVD, PSV ratio, CIMT, MFV, and PI, blue line) are shown. *indicates statistically significant (p<0.05) differences relative to classical cardiovascular risk scores (black lines). Abbreviations: ROC, receiver operating characteristics; FSRS, Framingham stroke risk score; PSV, peak-systolic velocity; CIMT, common carotid artery intima-media thickness; MFV, mean flow velocity; PI, pulsatility index; PSV/EDV ratio, peak-systolic-and-end-diastolic-velocity ratio; ASCVD, atherosclerotic cardiovascular disease.

**Fig 3:**
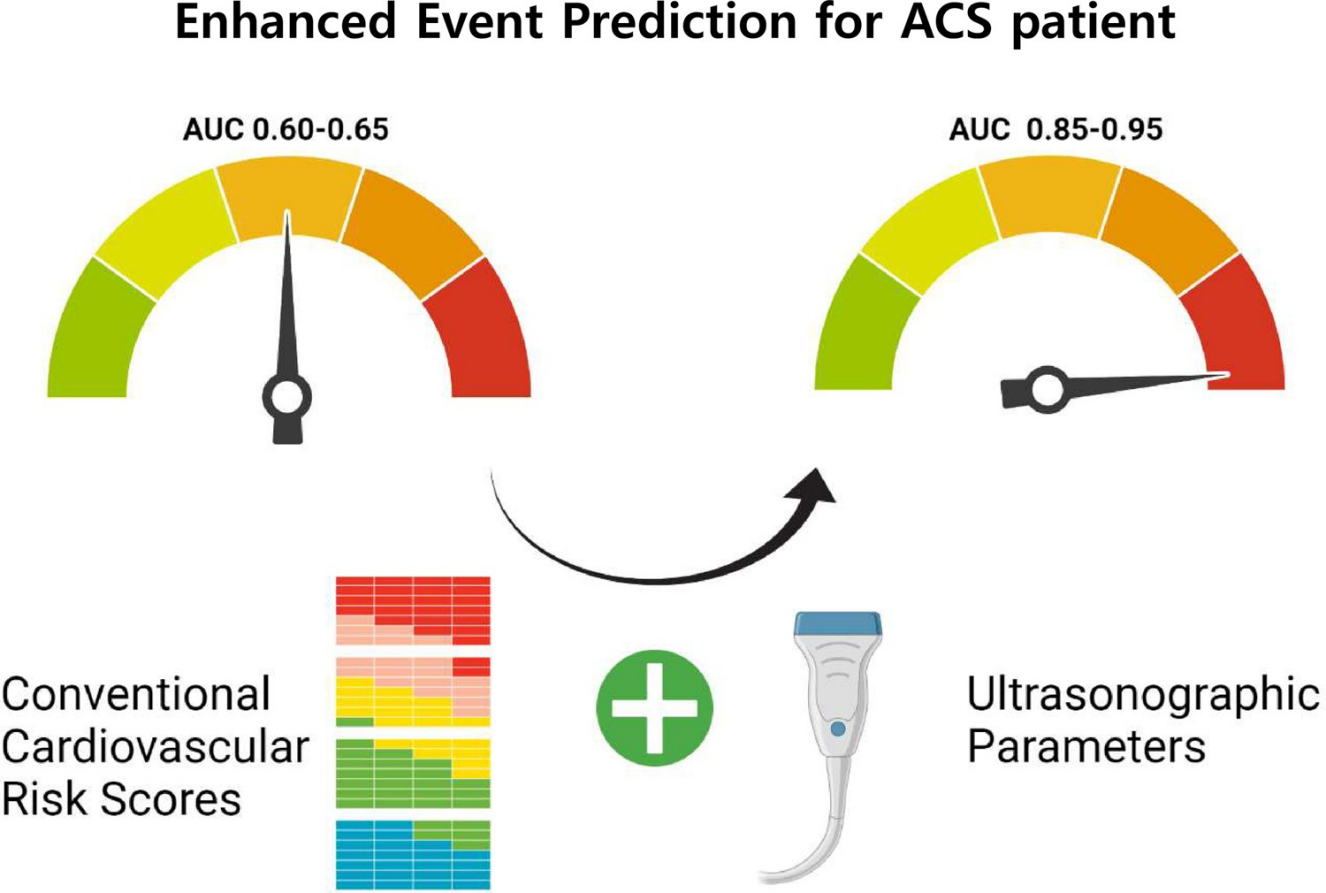
A graphical illustration of key findings. The improvement of predictive performance of the novel risk prediction models for asymptomatic carotid stenosis using combination with ultrasonographic parameters and conventional cardiovascular risk scores. Abbreviations: ACS, asymptomatic carotid stenosis.

In the Cox regression analysis, the C-statistics of the FSRS and ASCVD risk score were 0.646 and 0.649, respectively, for the prediction of relevant IS. For the prediction of composite outcomes, the C-statistics of the FSRS and ASCVD risk scores were 0.612 and 0.649, respectively. By adding the ultrasonographic parameters to the conventional cardiovascular risk scores, the C-statistics of the novel risk prediction models were significantly improved and decreased in the Akaike information criterion for the prediction of relevant IS and composite outcomes. The top three models are presented in Table 3 and Figs 1 and 2, respectively. Generally, models with C-statistics were composed of conventional cardiovascular risk scores, parameters of carotid stenosis degree, CIMT, plaque characteristics, and PI. For prediction of relevant IS, C-statistics of the FSRS and ASCVD risk scores combined with ultrasonographic parameters increased up to 0.937 from 0.646 (p<0.001) and 0.941 from 0.649 (p<0.001), respectively. For the prediction of composite outcomes, the C-statistics of the FSRS and ASCVD risk scores increased 0.856 from 0.612 (p<0.001) and 0.886 from 0.649 (p<0.001) by adding ultrasonographic values, respectively. A graphical illustration of the key findings is presented in Fig 3.

**Table 3:** Adjusted AUC for different prediction models with comparison to conventional risk scores only models.

Risk Prediction Model	C-statistics	AIC	p-value for difference in C-statistics
**For Relevant Ischemic Stroke**			
**FSRS only**	0.646	82.6	Reference
**Model 1: FSRS + PSV + CIMT + high-risk plaque**^a^ **+ PI**	0.937	59.6	<0.001*
**Model 2: FSRS + PSV ratio+ CIMT + high-risk plaque + PI**	0.927	63.6	<0.001*
**Model 3: FSRS + PSV/EDV ratio + CIMT + high-risk plaque + PI**	0.924	64.2	<0.001*
**ASCVD risk score only**	0.649	83.0	Reference
**Model 1: ASCVD+ PSV + CIMT + high-risk plaque + PI**	0.941	59.1	<0.001*
**Model 2: ASCVD + PSV/EDV ratio + CIMT + high-risk plaque + PI**	0.928	63.7	<0.001*
**Model 3: ASCVD + PSV ratio + CIMT + high-risk plaque + PI**	0.926	62.0	<0.001*
**For Composite Outcomes**			
**FSRS only**	0.612	130.4	References
**Model 1: FSRS + PSV ratio + CIMT + high-risk plaque**^a^ **+ Collateral flow + MFV + PI**	0.856	99.1	<0.001*
**Model 2: FSRS + PSV/EDV ratio + CIMT + high-risk plaque + Collateral flow + MFV+ PI**	0.855	98.3	<0.001*
**Model 3: FSRS + PSV + CIMT + high-risk plaque + Collateral flow + PI**	0.854	94.5	<0.001*
**ASCVD risk score only**	0.649	83.0	References
**Model 1: ASCVD+ PSV + CIMT + high-risk plaque + PI**	0.886	59.1	<0.001*
**Model 2: ASCVD + PSV/EDV ratio + CIMT + MFV + PI**	0.882	63.7	<0.001*
**Model 3: ASCVD + PSV ratio + CIMT + MFV+ PI**	0.881	62.0	<0.001*

Abbreviations: AUC, area under the curve; AIC, Akaike information criterion; FSRS, Framingham Stroke Risk Score; CIMT, carotid intima-media thickness; St Mary’s ratio, peak-systolic velocity to end-diastolic velocity ratio; MFV, mean flow velocities; PSV, peak-systolic velocity; ASCVD, atherosclerotic cardiovascular disease.

* indicates a significant difference (p<0.05).

^a^ hypoechoic or ulcerative plaque.

## Discussion

To the best of our knowledge, this is the first study to validate the predictive performance of conventional cardiovascular risk scores (FSRS and ASCVD risk scores) in the ACS population. In addition, this study showed that the combination of ultrasonographic parameters with conventional cardiovascular risk scores showed excellent performance in the prediction of stroke and composite outcome in ACS patients compared to the moderate performance of the model using only conventional cardiovascular risk scores with a statistically significant difference of p<0.001. Our data reinforce the role of ultrasonography in identifying high-risk of further vascular events for patients with ACS in primary practice.

### The role of conventional scores in predicting stroke in ACS population

From the observation study of multiple cohorts, 2.4% of the general population had moderate ACS [26]. Annually, approximately 3% of patients with ACS had stroke, which results in irreversible neurological sequelae and socioeconomic burden [27]. Therefore, it is critical to identify the high-risk population within asymptomatic individuals. The FRS and ASCVD risk scores are the most well-regarded risk prediction models for cardiovascular and cerebrovascular diseases although they have not yet been validated in ACS. Our data showed that FSRS and ASCVD scores had moderate predictive power for relevant ischemic stroke and composite outcomes in patients with ACS (C-statistics of 0.612 and 0.649, respectively). The overall performance corresponds with previous studies reporting C-statistics of 0.65 to 0.73 for ASCVD risk score for all CVDs and 0.65 for the FSRS for incident stroke in various subsets [28–30]. In our results, the C-statistics of the ASCVD risk score were higher compared to that of the FSRS in original or novel risk prediction models combining the FSRS or ASCVD risk scores with ultrasonographic parameters. FSRS was developed in a Caucasian population cohort. The ASCVD risk score, which was later developed, emphasized other races, cholesterol status, and stroke as outcomes [1]. In this context, the ASCVD risk score may have a higher performance compared to the FSRS for predicting stroke and composite outcomes in patients with nonwhite populations, as in this Korean study. Overall, our results imply that the conventional cardiovascular risk scores of the FSRS and ASCVD may also play a role in the prediction of stroke in the ACS population.

### Combining additional ultrasonographic parameters with conventional cardiovascular risk scores

However, the performance of conventional cardiovascular risk scores is relatively poor compared to previous studies, and the prediction performance for stroke may require improvement [30–33]. From this perspective, additional factors that could improve the predictive power have been studied, including high-sensitivity C-reactive protein, CIMT, and coronary calcium score [34–37]. In these studies, the most highly achieved C-statistics was 0.75, and the maximum increment of C-statistics by the additional factors was 0.15.

Considering the significance of atherosclerotic burden or hemodynamic status in ACS, a combination of hemodynamic ultrasonographic parameters presents a rational strategy for predicting stroke in ACS. Therefore, the introduction of cardiovascular surrogate markers and hemodynamic parameters could provide a reasonable explanation for the high accuracy of novel risk prediction models for predicting stroke and composite outcomes. Recently, measuring plaque, CIMT, PSV value, PSV ratio, and PSV/EDV ratio by an annual scan was thought to have added value in ACS because it determined individuals to adhere to aggressive prevention therapy or progress to interventional therapy [38–40]. According to the atherosclerosis risk in communities (ARIC) study, Ballantyne et al. revealed that the performance of conventional cardiovascular risk scores in predicting CVD could be improved by adding plaque data and CIMT to the FRS model [41, 42]. Similarly, our results demonstrated that the combination of ultrasonographic parameters significantly improved the prediction performance for relevant IS and composite outcomes, as shown in Fig 3 [43, 44]. Given the considerable incidence of events (8.0% of ischemic stroke/TIA and 18.0% of composite outcomes), the prediction of risk in ACS patients is clinically important.

The most important clinical application of our study is successful identification of high-risk patients with ACS that should be given more attention from clinicians in the clinical setting. Recently introduced techniques, including plaque imaging, have shown excellent performance in stratifying high-risk patients, although, it requires expensive equipment and setup. On the other hand, TCD is noninvasive and cost-effective, has excellent accessibility, and is available in the majority of stroke clinics worldwide. Therefore, our results have the potential to be applied to a larger number of patients. Though it is important to observe the clinical benefit of the model in a prospective cohort from the detection of high-risk patients, optimization of therapy (including intervention), and improvement in clinical outcomes.

It must be noted that the PSV/EDV ratio correlated with the relevant IS and PI of the MCA with composite outcomes, as shown in Table 2. The PSV/EDV ratio is known to be a precise and reliable hemodynamic marker, especially in cases of severe stenosis [45–47]. Because our patients had a relatively old age (mean age 65.6 years) and the mean PSV value was 194 cm/s, which corresponded to the degree of ICA stenosis of 60% –70%, the PSV/EDV ratio would be more predictive of stroke in ACS. PI, which is related to cerebral hemodynamics and cerebral arterial stiffness, was significantly associated with composite outcomes [48, 49]. Based on these results, we suggest that PI might reflect increased arterial stiffness in systemic circulation as well as in cerebral circulation. However, their correlation was marginally significant in the univariate analysis, and the results of the multivariate analysis showed that the effect of PI was not significant in the prediction of composite outcomes in ACS.

### Limitations

This study has several limitations. First, the number of included patients was relatively small (150), which may have resulted in low statistical power and a lesser effect of ultrasonographic addition. Second, the median 5-year follow-up period was relatively short to identify the occurrence of stroke or composite outcomes. In this study, CIMT and the presence of high-risk plaques were not significantly associated with relevant stroke in the Cox regression owing to the small number of relevant ischemic stroke events. However, the incidence of 36 events (12 strokes and 24 composite outcomes) over a mean 5-year period was similar to that reported previously [7, 50, 51]. Therefore, our results are reliable despite the small number of events. Third, longitudinal changes in baseline clinical information and ultrasonographic parameters were not analyzed. In the future, a large-scale prospective cohort study to predict stroke risk should be performed.

## Conclusions

In summary, our data suggests that the application of the FSRS and ASCVD risk scores is feasible in patients with ACS for the prediction of IS and CVD, and the addition of ultrasonographic parameters improves the predictive power of conventional cardiovascular risk scores. In primary practice, these novel risk prediction models could help to better identify high-risk patients with ACS who should be given more clinical attention.

## Supporting information

S1 Fig Selection of patients through the inclusion and exclusion criteria. Abbreviations: SBP, systolic blood pressure; DBP, diastolic blood pressure; HDL, high-density lipoprotein; ECG, electrocardiogram; ASCVD, atherosclerotic cardiovascular disease, MRI, magnetic resonance imaging; TCD, transcranial Doppler ultrasonography, CDU, carotid duplex ultrasonography.
(PDF)

S1 Table The database set used for the study. All relevant data are included in the manuscript and its supporting information files.
(XLSX)
