# Supplementary material for: Cardiovascular risk scores in asymptomatic carotid stenosis: A validation study with ultrasonographic parameters
Source: PLoS One. 2022 Apr 1;17(4):e0265732. doi: 10.1371/journal.pone.0265732 (PMC8975163; doi:10.1371/journal.pone.0265732)
Supplement: S1 Fig — Abbreviations: SBP, systolic blood pressure; DBP, diastolic blood pressure; HDL, high-density lipoprotein; ECG, electrocardiogram; ASCVD, atherosclerotic cardiovascular disease, MRI, magnetic resonance imaging; TCD, transcranial Doppler ultrasonography, CDU, carotid duplex ultrasonography. (PDF) [file pone.0265732.s001.pdf]

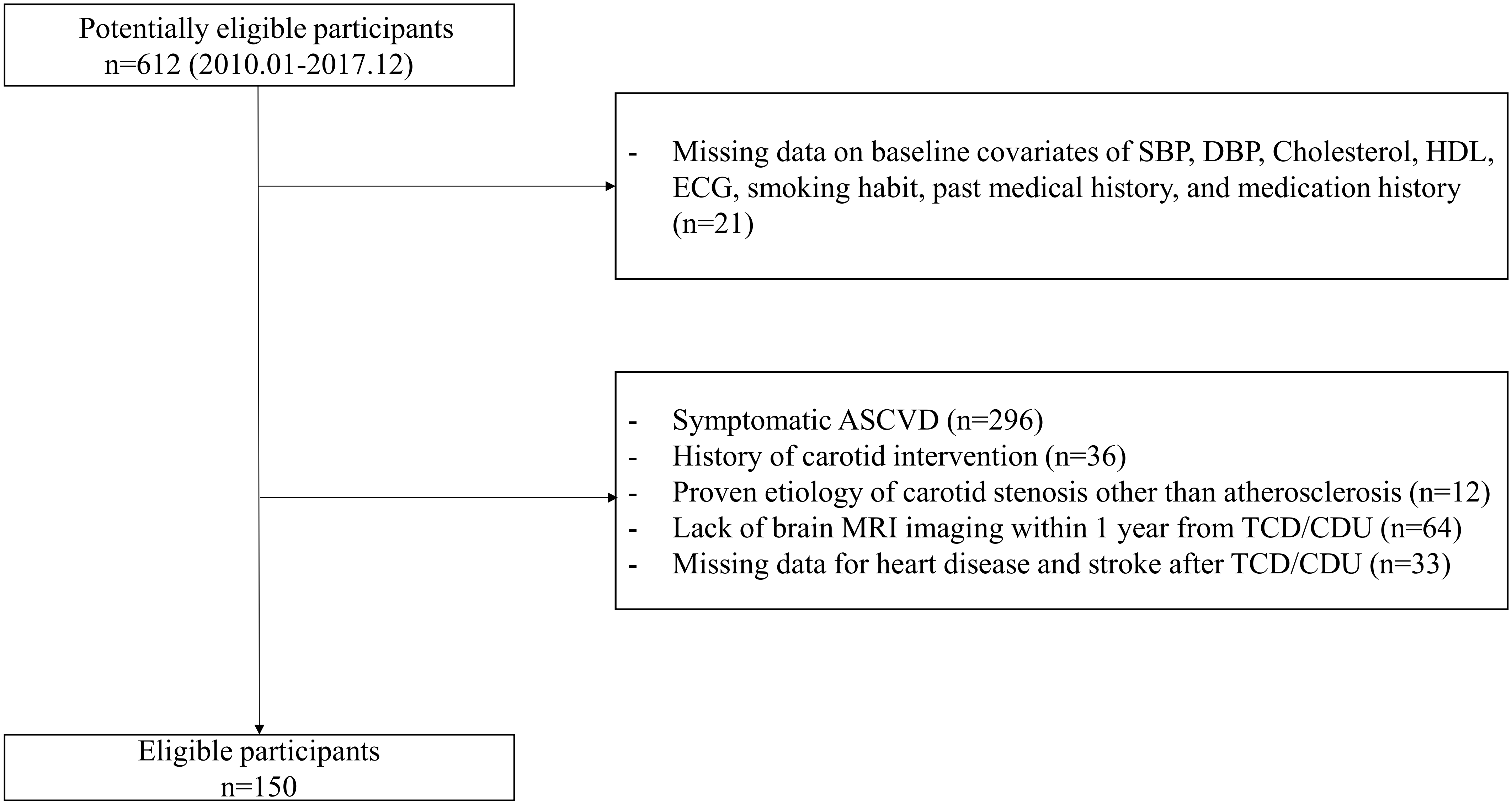
Potentially eligible participants
n=612 (2010.01-2017.12)
- Missing data on baseline covariates of SBP, DBP, Cholesterol, HDL, ECG, smoking habit, past medical history, and medication history (n=21)
- Symptomatic ASCVD (n=296)
- History of carotid intervention (n=36)
- Proven etiology of carotid stenosis other than atherosclerosis (n=12)
- Lack of brain MRI imaging within 1 year from TCD/CDU (n=64)
- Missing data for heart disease and stroke after TCD/CDU (n=33)
Eligible participants
n=150
